# Comparison of Reactive Sites in 2(1*H*)-Quinolone Derivatives for the Detection of Biologically Important Sulfur Compounds

**DOI:** 10.3390/molecules28165965

**Published:** 2023-08-09

**Authors:** Jolanta Kolińska, Aleksandra Grzelakowska, Marcin Szala, Radosław Podsiadły

**Affiliations:** Institute of Polymer and Dye Technology, Faculty of Chemistry, Lodz University of Technology, Stefanowskiego 16, 90-537 Lodz, Poland; aleksandra.grzelakowska@p.lodz.pl (A.G.); marcin.szala@p.lodz.pl (M.S.); radoslaw.podsiadly@p.lodz.pl (R.P.)

**Keywords:** fluorescent probe, 2(1*H*)-quinolone, reactive sulfur species

## Abstract

Novel fluorescent probes based on 2(1*H*)-quinolone skeleton containing a malonate group (**Q1**–**Q3**) were synthesized and proposed for biothiols detection. Their chemical reactivity toward thiols was compared to the reactivity of derivative having a dicyanovinyl group (**Q4**) as a reactive site. The detailed photophysical properties of these compounds were assessed through the determination of absorption and fluorescence spectra, fluorescence quantum yield, and fluorescence lifetime. In the presence of biothiols, an increase in the fluorescence intensity of compounds **Q1**–**Q3** and a hypsochromic shift in their emission bands were observed. In contrast, the compound with the dicyanovinyl group (**Q4**) in the presence of biothiols and cyanide ion showed the quenching of fluorescence, while a fluorescence “turn on” effect was observed toward reactive sulfur species.

## 1. Introduction

In recent years, the interest in research on the biological role of sulfur species has increased significantly due to the importance of thiol compounds in both physiological and pathological processes [1,2,3,4]. Thiols that play an essential role in the biochemical processes of the cells include low-molecular compounds such as L-cysteine (L-Cys), L-glutathione (L-GSH), and *N*-acetyl-L-cysteine (L-ACC). The presence of a sulfhydryl group in thiol compounds affects their antioxidative properties as well as their high reactivity and affinity for heavy metals [5,6,7].

Peroxidative damage associated with the industrialization of the environment has been increasingly implicated as a cause of the rising incidence of cardiovascular disease, cancer, and neurodegenerative disorders [8,9]. For this reason, particular attention has been paid to the involvement of individual thiols in the detoxification processes of reactive oxygen species and electrophilic xenobiotics [10]. Changes in the levels of individual thiols in human plasma are observed in various pathological states. The change in the concentration and redox status of one of the thiols significantly affects the concentrations and the redox status of the remaining thiols. This can influence the activity of enzymes and the biological functions of the receptors, structural proteins, and antioxidant defenses. Therefore, it is crucial to determine the thiol redox status in physiological processes.

L-Cys belongs to amino acids containing the sulfhydryl group, which can be an active site in proteins and enzymes, for example, thiol proteinases. Despite its physiological neurotoxicity, L-Cys is necessary [11,12]. In cells, L-Cys is present at a very low level, while in the extracellular space, it is present mainly in the form of L-cystine [13]. Compared to the thiol tripeptide L-GSH, L-Cys is a less effective antioxidant and a much more dangerous pro-oxidant. The possibility of complex binding of metal ions confirms the antioxidant activity of L-Cys [14].

L-GSH is one of the most important antioxidants in the human body, which is responsible for oxidative homeostasis in cells [15,16,17]. The presence of L-GSH is necessary in cell division, in the regulation of intracellular metabolism and apoptosis [18,19]. Many factors affect L-GSH reduction. The most common are stress, incorrect diet, environmental factors, infections, genetic defects, and metabolic disorders. Low L-GSH level leads to various pathological states [20].

L-ACC, the *N*-acetylated precursor of L-Cys, is an endogenous sulfuric amino acid that occurs naturally in the human body. L-ACC has strong antioxidant properties [21]. As a result, L-ACC is a popular compound with mucolytic properties, commonly used in treating upper respiratory illnesses and other disorders [22,23]. The presence of sulfhydryl groups makes this compound an excellent candidate for L-GSH reduction and it is capable of binding transition metal ions and heavy metal ions. In addition, L-ACC also exhibits the ability to directly scavenge free radicals, e.g., hydroxyl radicals (•OH) [24,25].

The current literature is overflowing with fluorescent probes for detecting biothiols that work via different reaction mechanisms. Among the most important mechanisms are the Michael addition, cyclization with aldehyde, and other reactions [26,27,28,29]. The introduction of appropriate electron-withdrawing groups to the chromophore system changes the properties of the fluorescent probe. The presence of a dicyanovinyl moiety in the structure of the probe causes a redshift in the emission band and increases the value of Stokes shift. A number of fluorophores containing a dicyanovinyl moiety have been described in the literature as fluorescent probes for the detection of various nucleophiles, including biothiols [30,31], reactive sulfur species (RSS) [32], and cyanide anion [33]. Another example of an electron-withdrawing group is the malonate moiety, which can quench the fluorescence of fluorophore. It is a more selective moiety because it is only applicable as the recognition site for the detection of biothiols [34,35,36,37].

The 2(1*H*)-quinolone fluorophore was selected because of its desirable photophysical properties [38]. In addition, quinolones are important compounds due to their wide range of biological activities [39], and they can be easily modified via alkylation at the amide nitrogen, making them suitable as fluorescent probes [40,41,42].

In our previous studies, we have synthesized, characterized, and used the derivatives of 2(1*H*)-quinolone with dicyanovinyl group as fluorescent sensors for thiols [30,31] and cyanide anion [33]. In the presence of the studied analyte, these compounds exhibit fluorescence quenching (on-off) as the detection signal.

**Scheme 1:**
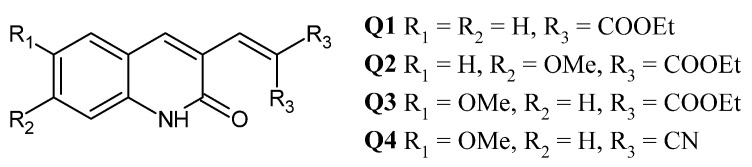
The chemical structure of studied compounds **Q1**–**Q4**.

Herein, we synthesized and characterized the fluorescent probes based on a 2(1*H*)-quinolone skeleton with two different reactive moieties. Their reactivity toward biothiols was compared. These compounds contain a malonate moiety (**Q1**–**Q3**) and a dicyanovinyl group (**Q4**) acting as an acceptor. The structures of the studied compounds are shown in Scheme 1.

## 2. Results

### 2.1. Synthesis and Characterization of ***Q1**–**Q3***

**Scheme 2:**
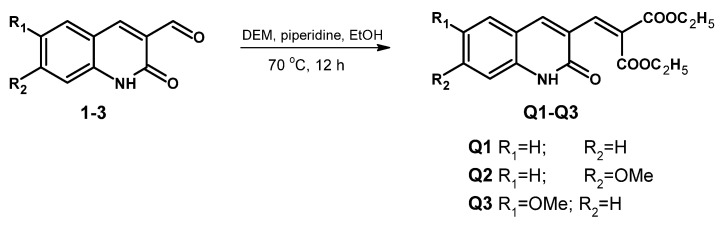
Synthesis of 2(1*H*)-quinolone derivatives, **Q1**–**Q3**.

**Figure 1:**
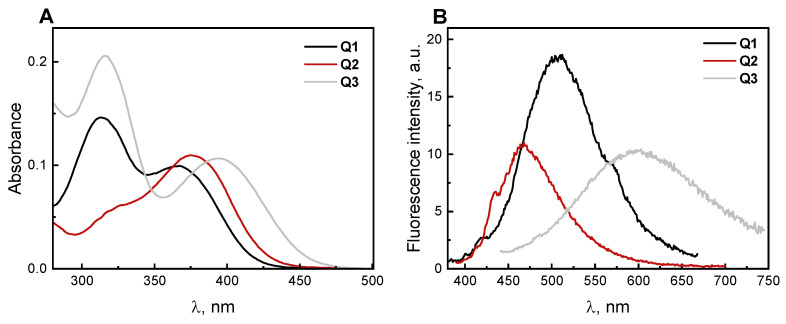
(**A**) The absorption and (**B**) fluorescence spectra of **Q1** (10 µM), **Q2** (6 µM), and **Q3** (10 µM) in a 0.1 M phosphate buffer with CH_3_CN (20%, *v*/*v*, pH = 7.4).

The novel derivatives of 2(1*H*)-quinolone, **Q1**–**Q3,** were obtained as results of the presented syntheses in Scheme 2. The appropriate three formyl derivatives of 2(1*H*)-quinolone (1-3) were earlier described in the literature [31,32]. They were condensed with diethyl malonate (DEM) in the presence of piperidine as a catalyst in anhydrous ethanol, yielding the compounds **Q1**–**Q3**. The chemical structures of **Q1**–**Q3** were confirmed via proton nuclear magnetic resonance (^1^H NMR) and electrospray ionization mass spectrometry (ESI MS) (Figures S1–S6). The obtained data agreed with the established structure of the molecules. The photophysical and spectroscopic properties of the novel compounds **Q1**–**Q3** were determined based on the recorded absorption and emission spectra. Detailed characteristics of the obtained compounds **Q1**–**Q3** are included in Table 1. Moreover, electronic absorption and emission spectra of **Q1**–**Q3** compounds are provided in Figure 1.

Compounds **Q1**–**Q3** exhibit two absorption bands located in the ultraviolet and visible ranges. Their absorption maximum (λ_abs_) is located in the range of 366–402 nm, while the emission maximum (λ_em_) is in the range of 460–600 nm. The presence of the methoxy substituent at position 6 in the quinolone skeleton significantly affects the spectroscopic properties of **Q3**. This compound has a huge shift in λ_em_ and λ_abs_ toward longer wavelengths compared to the unsubstituted derivative (**Q1**). Moreover, a small bathochromic effect is also observed for the methoxy derivative (**Q2**). **Q2** possesses the highest molar extinction coefficient value compared with other derivatives (**Q1** and **Q3**). Otherwise, the derivative **Q3** is characterized by a Stokes shift above 100 nm, suggesting that the ground and excited state geometry is different. In comparison, the values below 100 nm indicate that the geometry of these two states are similar (**Q1** and **Q2**). Additionally, the fluorescence quantum yield (Φ_em_) and the lifetime of the singlet excited state (τ) of compound **Q3** are also higher than Φ_em_ and τ of derivatives **Q1** and **Q2**, whereas the introduction of a dicyanovinyl group into the quinolone structure (**Q4**) causes an even larger bathochromic shift (λ_em_ and λ_abs_) and the highest values for Φ_em_ and τ.

**Figure 2:**
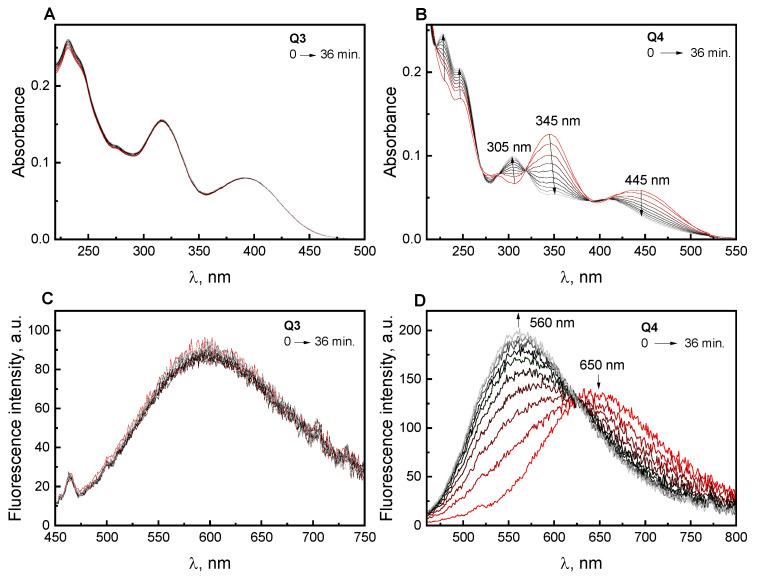
Changes in the absorption (**A**,**B**) and emission (**C**,**D**) spectra of **Q3** (10 μM) and **Q4** (20 μM) over time in a 0.1 M phosphate buffer with CH_3_CN (20%, *v*/*v*, pH = 7.4).

Our earlier paper [30,31] has shown that the 2(1*H*)-quinolone derivatives with a dicyanovinyl moiety are unstable in an aqueous medium and probably undergo hydrolysis. To compare the stability of **Q3** with **Q4**, their absorption and emission spectra were recorded in a 0.1 M phosphate buffer with CH_3_CN (20%, *v*/*v*, pH = 7.4) over 36 min. As shown in Figure 2, compound **Q3** is stable under these conditions, in contrast to the derivative **Q4**. It is evident that a compound with a malonate moiety (**Q3**) will be more suitable for potential use as a sensor for detecting biothiols.

Because of the observed difference in fluorescence of compounds with a malonate moiety (**Q1**–**Q3**) and the compound containing dicyanovinyl moiety (**Q4**), further investigations were carried out to explain the quenching mechanism acting in these compounds. The fluorescence decay profile of compounds **Q1**–**Q3** and **Q4** are presented in Figure S7 in acetonitrile with an excitation at 376.2 nm. Quantum yield of fluorescence (Φ_em_) and fluorescence lifetime (τ) are determined by the ratio between the rate constants of radiative (k_r_) and non-radiative (k_nr_) intramolecular decay processes [43,44]. From the presented data in Table S1, it is clear that the increase in fluorescence lifetime is due to a decrease in non-radiative decay. The k_r_/k_nr_ = 0.1025 obtained for compound **Q4** compared to the k_r_/k_nr_ in the range 0.0019–0.0245 for compounds **Q1**–**Q3**, indicates that a radiative pathway, which is responsible for fluorescence, is more favorable in **Q4**. Malonate moiety in the structure of 2(1*H*)-quinolone (**Q1**–**Q3**) causes the ICT process and a non-radiative pathway to dominate.

### 2.2. Absorption and Fluorescence Responses of ***Q1**–**Q3*** toward the Thiols

**Figure 3:**
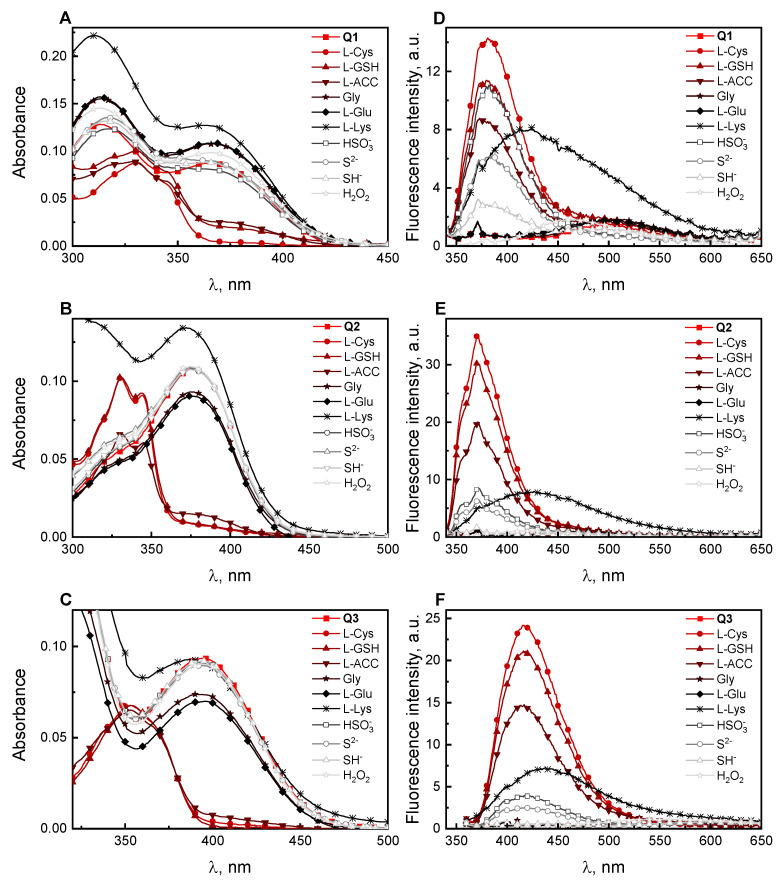
Absorption (**A**–**C**) and emission (**D**–**F**) spectra of compounds **Q1** (10 μM), **Q2** (6 μM), and **Q3** (10 μM) in the presence of various analytes (1 mM) in a 0.1 M phosphate buffer with CH_3_CN (20%, *v*/*v*, pH = 7.4).

The reactivity of **Q1**–**Q3** derivatives toward biothiols (L-Cys, L-GSH, and L-ACC), non-thiol amino acids (L-Glu, Gly, and L-Lys), and representative anions, including reactive sulfur species (HSO_3_^−^, SH^−^, and S^2−^), and reactive oxygen species (H_2_O_2_) were tested and the results are shown in Figure 3. The measurements were carried out in a 0.1 M phosphate buffer with CH_3_CN (20%, *v*/*v*, and pH = 7.4). The absorption spectra recorded in the presence of various analytes for **Q1**–**Q3** derivatives are presented in Figure 3A–C, and emission spectra are presented in Figure 3D–F, respectively. For compounds **Q1**–**Q3**, in the presence of biothiols (L-Cys, L-ACC, and L-GSH), the disappearance of the absorption band in the visible spectrum and the formation of a new band in the near ultraviolet range were observed. The absorption spectra for **Q1**–**Q3** derivatives in the presence of L-Cys and L-GSH over time (0–30 min.) are presented in Figure S8. The location of the maximum of a new absorption band for compounds **Q1**–**Q3** in the presence of biothiols depends on their chemical structure. For compounds **Q1** and **Q2**, the absorption maximum is located at 330 nm, and for **Q3** at 354 nm. In the presence of non-thiol amino acids (L-Glu, Gly, L-Lys) and the other analytes, absorption spectra of **Q1**–**Q3** did not display any significant changes. As shown in Figure 3D–F, fluorescence intensity at wavelengths 380, 370, and 415 nm increased in the presence of biothiols for **Q1**, **Q2**, and **Q3**, respectively. The highest increase in fluorescence intensity was recorded for L-Cys, L-GSH, and L-ACC. Moreover, a slight increase in fluorescence was also seen for HSO_3_^−^ and S^2−^.

**Figure 4:**
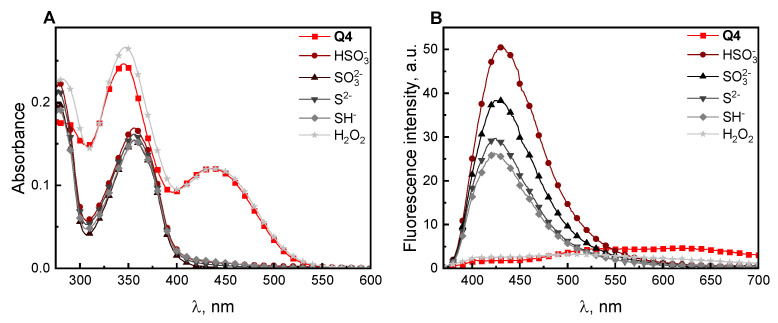
Absorption (**A**) and emission (**B**) spectra of compound **Q4** (20 μM) in the presence of reactive sulfur species and reactive oxygen species (1 mM) in a 0.1 M phosphate buffer with CH_3_CN (20%, *v*/*v*, pH = 7.4).

As shown in our earlier publications [30,33], the fluorescence of **Q4** was completely quenched in the presence of L-Cys or cyanide anion. Similar changes in the absorption spectra for all compounds (**Q1**–**Q4**) toward biothiols were observed. Here, to study the reactivity of the **Q4** probe in more detail, the absorption and emission spectra of **Q4** in the presence of reactive sulfur species (HSO_3_^−^, SO_3_^2−^, SH^−^, and S^2−^) and reactive oxygen species (H_2_O_2_) were also measured. The analysis of the absorption and emission spectra changes shows that RSS can interact with compound **Q4**. In their presence, a complete disappearance of the absorption band in the visible range of the spectrum and a formation of a new band with a maximum located in the near ultraviolet range were observed (Figure 4A). An analogous effect was observed in the case of emission spectra. As can be seen in Figure 4B, adding RSS to the compound of **Q4** causes the formation of a new emission band at 430 nm, whereas in the presence of H_2_O_2_, there were no changes in the position of the absorption and emission band maximum.

**Figure 5:**
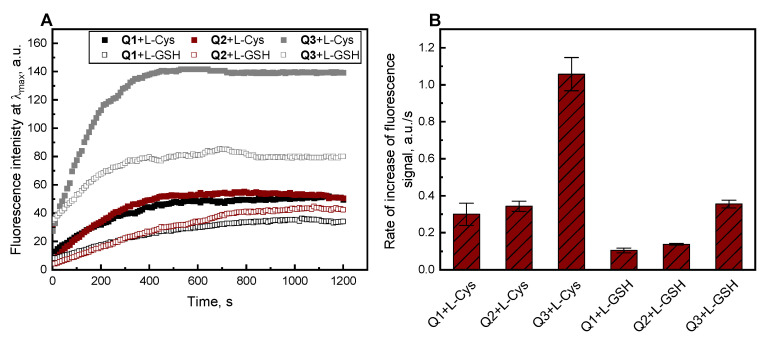
(**A**) Changes in fluorescence intensity of **Q1** (10 μM), **Q2** (6 μM), **Q3** (10 μM) in the presence L-Cys and L-GSH (100 μM) in a 0.1 M phosphate buffer with CH_3_CN (20%, *v*/*v*, pH = 7.4). (**B**) The rates of increase in the fluorescence signal intensity from **Q1**–**Q3** in the presence L-Cys and L-GSH.

The dependence of changes in the fluorescence intensity of **Q1**–**Q3** derivatives with time in the presence of a 10-fold excess of L-Cys and L-GSH in a 0.1 M phosphate buffer with CH_3_CN (20%, *v*/*v*, pH = 7.4) were also carried out, and the results are presented in Figure 5. The results of kinetic study showed that the fluorescence emission of compounds **Q1**–**Q3** increased and reached a maximum after about 400 s. Thus, from the experiment conducted, it follows that the fluorescence response of the tested derivatives to biothiols is obtained rapidly. The highest value of the rate of increase in the fluorescence signal intensity was observed for **Q3** in the presence of L-Cys.

**Figure 6:**
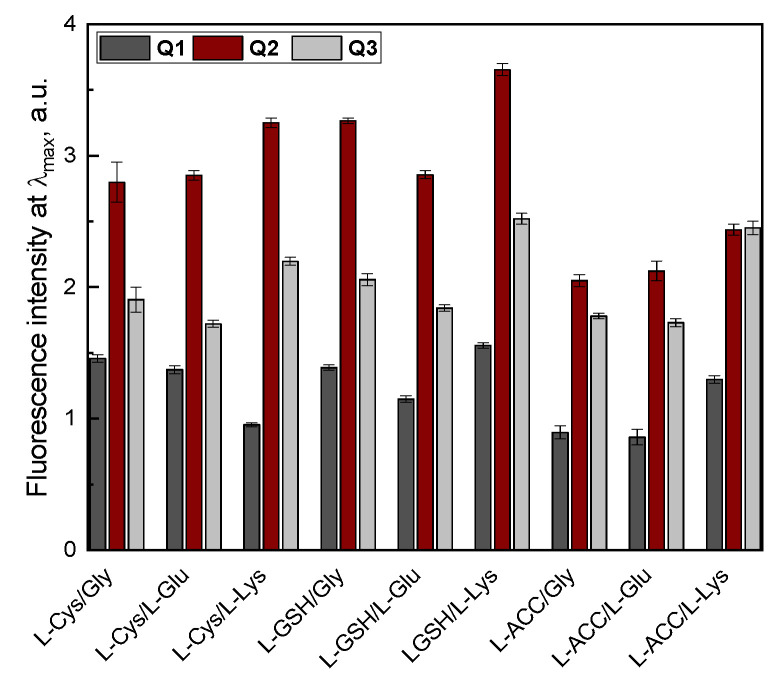
Fluorescence intensity of **Q1** (10 μM), **Q2** (6 μM), and **Q3** (10 μM) in a 0.1 M phosphate buffer with CH_3_CN (20%, *v*/*v*, pH = 7.4).

Furthermore, competitive selectivity studies of three biothiols with some representative amino acids (Gly, L-Glu, and L-Lys) were carried out. As illustrated in Figure 6, all of the compounds, **Q1**–**Q3,** demonstrated an ability to selectively detect the thiol group in the presence of other biologically relevant nucleophiles, such as the amino groups.

However, it is worth noting that the selectivity of **Q2** and **Q3** is significantly increased probably due to the presence of an electron-donating group in the quinolone structure.

**Figure 7:**
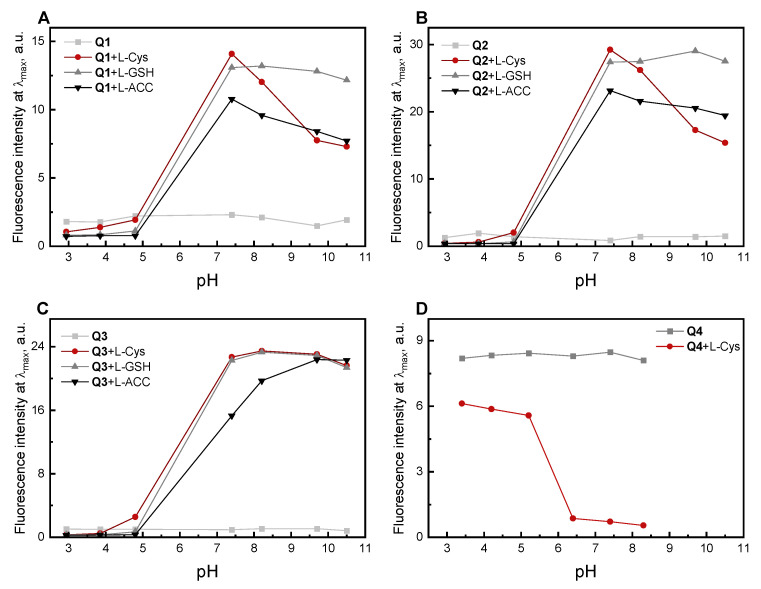
Fluorescence intensity of (**A**) **Q1** (10 μM), (**B**) **Q2** (6 μM), (**C**) **Q3** (10 μM), and (**D**) **Q4** (20 μM) at different pH values in the absence/presence of biothiols (1 mM).

The pH value significantly influences the spectroscopic properties and the optical response of the chemosensors in the presence of analytes. Therefore, the impact of pH on the fluorescence intensity of compounds **Q1**–**Q3** relative to L-Cys, L-GSH, and L-ACC in solutions of different pH (3.1–10.5) was studied (Figure 7A–C). In the absence of biothiols, the compounds **Q1**–**Q3** displayed minimal emission over all analyzed pH range at 508 nm, 468 nm, and 600 nm, respectively, indicating good stability of these derivatives in the broad pH range. The fluorescence intensities of **Q1**–**Q3** in the presence of biothiols were pH dependent. Compounds **Q1**–**Q3** at a pH 3.1–5.0 show negligible fluorescence in the presence of biothiols, while the highest fluorescence intensity after adding thiols was observed at pH 7.4. However, above this value, the fluorescence intensity gradually decreased. This proves that the most active form of thiols is the nucleophilic form (thiolate anion). This result implies that derivatives **Q1**–**Q3** can potentially detect biothiols under physiological conditions (at a pH of 7.4). For comparison, the impact of pH on the fluorescence intensity of compound with a dicyanovinyl group, **Q4,** on the detection of L-Cys is presented in Figure 7D. The compound **Q4**, in contrast to the **Q1**–**Q3** derivatives, showed a decrease in the fluorescence intensity at a pH above 6.5. Additionally, the influence of pH value on the detection of HSO_3_^−^ via **Q4** was investigated. As can be seen in Figure S9, an increase in the fluorescence intensity after adding HSO_3_^−^ in the pH range from 2.6 to 7.5 is observed. This test showed that the **Q4** derivative is most suitable for detecting this reactive sulfur form in a wide pH range.

**Scheme 3:**
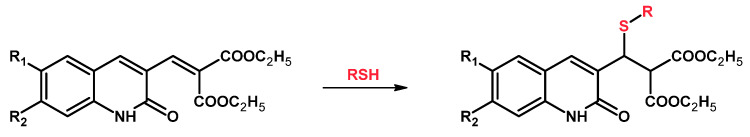
The proposed reaction of 2-mercaptoethanol with compounds **Q1**–**Q3**.

We propose that the reaction of compounds **Q1**–**Q3** and sulfhydryl compounds proceeds via nucleophilic Michael addition to the α,β-unsaturated double bond. 2-Mercaptoethanol (ME) was selected as the sulfhydryl compound for the study confirming the proposed reaction mechanism (Scheme 3). The product of the reaction of ME with compound **Q3** was confirmed via proton nuclear magnetic resonance (^1^H NMR) spectroscopy. In the aromatic part of the spectrum, the signal corresponding to the double bond in the malonate moiety of compound **Q3** disappeared, and two new signals at 2.74 and 4.72 ppm appeared in the aliphatic part (Figure S10). Moreover, the product of the reaction of **Q3** and ME was characterized using mass spectroscopy. For this purpose, the mass spectrum was recorded at a 100-fold excess ME (Figure S11). The characteristic molecular ion peak at *m*/*z* = 446.1243 ([M + Na]^+^) corresponding to the expected adduct of **Q3**-**ME** (calculated for C_20_H_25_NO_7_SNa *m*/*z* = 446.1249) was observed in the mass spectrum. The obtained result demonstrated that the thiol compound (ME) could react with the unsaturated double bond of the compound **Q3** to yield product **Q3**-**ME** at a 100-fold excess ME.

Additionally, the emission spectra of compounds **Q1**–**Q3** were recorded in the presence of biothiols (L-Cys, L-GSH, and L-ACC) that were previously treated with a thiol-blocking agent (NEM) (Figure S12). The presence of NEM caused the blocking of the group thiol (−SH) in the biothiols [44,45,46]. As shown, biothiols treated with NEM did not enhance the fluorescence of the studied compounds **Q1**–**Q3**. This suggests that the fluorescent response toward thiols results from the probes’ reaction with the sulfhydryl group present in the amino acid structure.

**Figure 8:**
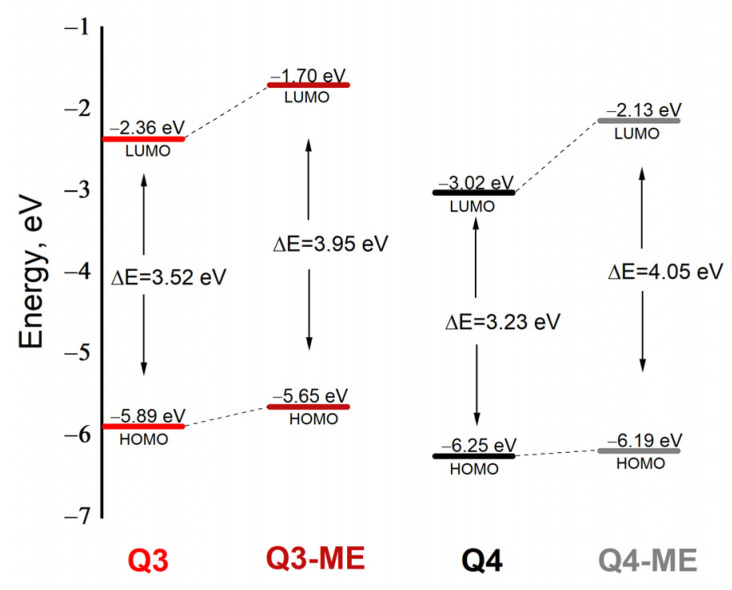
HOMO and LUMO orbitals of compounds **Q3** and **Q4** and the addition products with 2-mercaptoethanol (**Q3**-**ME** and **Q4**-**ME**).

The quantum chemical calculations were performed to understand the reasons for the enhancement or quenching of the fluorescence of compounds **Q1**–**Q3** and **Q4**, which contain two different moieties as reaction sites for biothiols. In the DFT calculation (B3LYP/6-31+G (d,p)) [47], the structures of the derivatives **Q3** and **Q4** and their products of the reaction with 2-mercaptoethanol (**Q3**-**ME** and **Q4**-**ME**) were optimized. The energy gap (ΔE) for compounds **Q3** and **Q4** between the HOMO (highest occupied molecular orbital) and the LUMO (lowest unoccupied molecular orbital) was found to be 3.52 and 3.23 eV, respectively. After the addition of 2-mercaptoethanol, the ΔE increased to 3.95 and 4.05 eV for compounds **Q3**-**ME** and **Q4**-**ME**, respectively. These differences are illustrated in Figure 8. The difference in the spectroscopic properties of the derivatives **Q3** and **Q4** is related to their energy gap values. Based on the obtained data, it can be unequivocally stated that the derivative with the malonate moiety (**Q3**) has a more significant energy gap. Therefore, according to the literature [48], it will have a lower fluorescence quantum yield. In contrast, the derivative with the dicyanovinyl moiety (**Q4**) has a lower energy gap and a higher quantum fluorescence yield. Moreover, comparing these compounds, it can be noticed that smaller the energy gap, the more significant the redshift in the emission. The small energy gap value characterizes the product of the reaction of **Q3** with ME (**Q3**–**ME**) and an increase in fluorescence intensity was observed. In contrast, the product **Q4**-**ME** is characterized by a more significant energy gap and fluorescence quenching after the reaction of **Q4** with thiols was observed.

## 3. Materials and Methods

### 3.1. General

The reagents and solvents were commercial products from Sigma-Aldrich (Poland) and were used directly without further purification. The course of the reaction and the purity of the obtained compounds were routinely checked using thin layer chromatography (TLC) on silica gel 60 F254 plates (Merck, Germany). Plates were visualized under UV light (λ = 254 nm or 365 nm). The crude probes were purified using column chromatography on silica gel 60 (0.063–0.200 mm, Merck, Germany). Melting points were checked on the Boeöthius melting point apparatus (type PHMK 05, Radebeul, Germany), and were uncorrected. Proton nuclear magnetic resonance (^1^H NMR) spectra of the previously unreported compounds were recorded on a Bruker Avance DPX 250 (Rheinstetten, Germany) spectrometer in deuterated solvent (dimethyl sulfoxide, DMSO-*d_6_*) with TMS as the internal standard. The chemical shifts (δ values) and coupling constants (*J* values) are expressed in parts per million (ppm) and hertz (Hz), respectively. NMR peak multiplicities are described as follows: *s* (singlet), *d* (doublet), *ddd* (doublet of doublets of doublets), *t* (triplet), *q* (quartet), *m* (multiplet), and *brs* (broad singlet). High-resolution mass spectrometry (HRMS) experiments were performed with a mass spectrometer equipped with an electrospray ionization (ESI) source operated in a positive ion mode and quadrupole-time-of-flight mass analyzer (Synapt G2-Si mass spectrometer, Waters). Data were identified as hydrogen ion adducts ([M + H]^+^).

### 3.2. Spectroscopic Measurements

UV-vis absorption and fluorescence spectra were recorded using a Jasco V-670 UV-vis/NIR spectrophotometer (Jasco, Japan) and an FLS-920 spectrofluorometer (Edinburgh Instruments, UK), respectively. In each case, quartz cuvettes (1 cm) were used. The excitation/emission wavelengths were 366/508 nm, 376/468 nm, 394/600 nm, and 440/636 nm for **Q1**, **Q2**, **Q3**, and **Q4**, respectively. The excitation and emission slits’ widths were 1.5 nm. The final concentration of the compounds in the solution corresponded to their absorbance of about 0.1 in λ_max_ and were 10, 6, 10, and 20 µM for **Q1**, **Q2**, **Q3**, and **Q4**, respectively. The pH values were determined with a CPI-551 microcomputer pH/ion meter (Elmetron, Poland).

Fluorescence quantum yields were determined via a comparative method using fluorescein as a reference compound [49]. The fluorescence lifetimes of the compounds were obtained as previously described [32]. Moreover, the radiative decay rate constant k_r_ and the non-radiative decay rate constant k_nr_ for compounds **Q1**–**Q4** were determined using the following Equations (1) and (2):

k_r_ = Φ_em_/τ (1)

k_nr_ = (1/τ) − k_r_ (2)

where Φ_em_ and τ are the fluorescence quantum yield and average fluorescence lifetime, respectively.

### 3.3. General Procedure Synthesis of Compound ***Q1**–**Q3***

Diethyl malonate (DEM, 1.2 mmol) was put in the solution of appropriate 3-formyl-2(1*H*)-quinolone derivative (1 mmol) in absolute ethanol (10 mL), and then two drops of piperidine were added. The resulting mixture was stirred at 70 °C for 12 h under the inert atmosphere. The progress of the reaction was monitored via TLC (eluent: CH_3_CN:CH_2_Cl_2_, 1:1, *v*/*v*). After completion, the mixture was cooled to room temperature and filtered to yield a solid product **Q1** (0.18 g; 58%; R_f_ = 0.77) with a melting point of 168–170 °C, ^1^H NMR (DMSO-*d_6_*, 250 MHz) *δ* 1.18 (*t*, *J* = 7.1 Hz, 3H), 1.26 (*t*, *J* = 7.1 Hz, 3H), 4.25 (*q*, *J* = 7.1 Hz, 4H), 7.11–7.26 (*m*, 1H), 7.28–7.38 (*m*, 1H), 7.59 (*ddd*, *J* = 8.4, 5.7, 1.5 Hz, 1H), 7.71 (*ddd*, *J* = 11.2, 8.9, 1.1 Hz, 1H), 7.75–7.95 (*m*, 1H), 8.05 (*s*, 1H), 12.17 (*brs*, 1H); HRMS (MS ESI) *m*/*z*: [M + H]^+^ calcd. for C_17_H_18_NO_5_ 316.1185, found 316.1186.

**Q2** (0.31 g; 90%; R_f_ = 0.80), melting point 183–185 °C, ^1^H NMR (DMSO-*d_6_*, 250 MHz) *δ* 1.19 (*t*, *J* = 7.1 Hz, 3H), 1.22 (*t*, *J* = 7.1 Hz, 3H), 3.83 (*s*, 3H), 4.22 (*q*, *J* = 7.1 Hz, 4H), 6.72–6.88 (*m*, 2H), 7.64 (*d*, *J* = 8.8 Hz, 1H), 7.75 (*d*, *J* = 0.8 Hz, 1H), 7.97 (*s*, 1H), 12.04 (*brs*, 1H); HRMS (MS ESI) *m*/*z*: [M + H]^+^ calcd. for C_18_H_20_NO_6_ 346.1291, found 346.1294.

**Q3** (0.25 g; 72%; R_f_ = 0.78), melting point 190–192 °C, ^1^H NMR (DMSO-*d_6_*, 250 MHz) *δ* 1.15 (*t*, *J* = 7.1 Hz, 3H), 1.23 (*t*, *J* = 7.1 Hz, 3H), 3.79 (*s*, 3H), 4.26 (*q*, *J* = 7.1 Hz, 4H), 7.18–7.27 (*m*, 3H), 7.77 (*d*, *J* = 0.8 Hz, 1H), 7.99 (*d*, *J* = 0.8 Hz, 1H), 12.08 (*brs*, 1H); HRMS (MS ESI) *m*/*z*: [M + H]^+^ calcd. for C_18_H_20_NO_6_ 346.1290, found 346.1291.

### 3.4. General Procedure Spectroscopic Experiments of Compounds ***Q1**–**Q4***

All spectroscopic measurements were performed in a 0.1 M phosphate buffer with acetonitrile (20%, *v*/*v*, pH = 7.4). The following buffers for pH measurements were used: an acetate buffer (0.1 M, for pH values ranging from 2.6 to 5.6), a phosphate buffer (0.1 M, for pH 6.4–8.3), and carbonate–bicarbonate buffer (0.1 M, for pH 9.5–10.5). Stock solutions of compounds **Q1**–**Q4** (1 mM) were prepared in acetonitrile (CH_3_CN). The following solutions (10 mM) were prepared in distilled water: biothiols (L-Cys, L-GSH, and L-ACC), non-thiol amino acids (L-Glu, Gly, and L-Lys), reactive sulfur species (HSO_3_^−^, SH^−^, and S^2−^), reactive oxygen species (H_2_O_2_) and the thiol-blocking agent, N-ethylmaleimide (NEM). The fluorescence and UV absorption spectra were recorded at an ambient temperature (24–25 °C).

### 3.5. Computational Methods

All the quantum chemical calculations were performed with the Gaussian 09 package [50,51]. Geometries of the compound **Q3** and **Q4** were optimized via Becke’s LYP (B3LYP) exchange–correlation functional with 6-31G (d, p) basis set based on density functional theory (DFT). These computations were carried out in a gas phase. A harmonic frequency analysis characterized the optimized structures as local minima. The time-dependent density functional theory (TD-DFT) at the B3LYP/6-31G ** level was used to calculate the electronic transition energies (HOMO and LUMO).

## 4. Conclusions

In summary, three compounds, **Q1**–**Q3**, based on a 2(1*H*)-quinolone skeleton with malonate moiety have been synthesized and characterized. These compounds exhibit a fluorescence response to the sulfhydryl group in biothiols (L-Cys, L-GSH, and L-ACC) in a 0.1 M phosphate buffer with CH_3_CN (20%, *v*/*v*, pH = 7.4). The results show that the reaction of **Q3** with thiols proceeds via the nucleophilic addition of the sulfhydryl group to the malonate moiety. The responses of compounds containing a malonate group (**Q1**–**Q3**) and a dicyanovinyl group (**Q4**) were compared with the tested sulfhydryl compounds. Our studies on variously substituted 2(1*H*)-quinolones allow us to conclude that the applied reactive system influences the interaction of the analyte with the sensor. Based on the observed reactivity of the compounds **Q1**–**Q4**, we conclude that the compounds possessing a malonate group in the structure (**Q1**–**Q3**) are more useful for the detection of biothiols (L-Cys, L-GSH, and L-ACC) because these compounds distinguish biothiols among reactive sulfur forms as opposed to the compound possessing a dicyanovinyl group (**Q4**), which is less selective toward reactive sulfur species.

## Figures and Tables

**Table 1:** Spectroscopic characterization of compounds **Q1**–**Q4**.

	Q1	Q2	Q3	Q4
λ_abs_ (nm)	372 ^a^	378 ^a^	402 ^a^	456 ^a^ [30]
	366 ^b^	376 ^b^	394 ^b^	
ε (M^−1^ × cm^−1^)	9700 ^a^	14,300 ^a^	7100 ^a^	7900 ^a^ [30]
λ_em_ (nm)	462 ^a^	460 ^a^	548 ^a^	625 ^a^ [30]
	508 ^b^	468 ^b^	600 ^b^	
Φ_em_ (%)	0.20 ^a^	0.19 ^a^	2.42 ^a^	9.3 ^a^ [30]
SS (nm)	90 ^a^	82 ^a^	146 ^a^	169 ^a^ [30]
	142 ^b^	92 ^b^	206 ^b^	
τ (ns)	1.25 ^a^	1.86 ^a^	3.09 ^a^	3.12 ^a^

^a^ CH_3_CN, ^b^ 0.1 M phosphate buffer with CH_3_CN (20%, *v*/*v*, pH = 7.4).

## Data Availability

The data presented in this study are available in the article and supplementary material.

## Supplementary Materials

The following supporting information can be downloaded at: https://www.mdpi.com/article/10.3390/molecules28165965/s1, Figures S1–S3 1H NMR spectra of **Q1**–**Q3** in DMSO-*d_6_*; Figures S4–S6 HRMS spectra of **Q1**–**Q3**; Figure S7 The fluorescence decay profiles of the compounds **Q1**–**Q4** in acetonitrile with excitation at 376.2 nm. Lamp response was determined using Ludox. The fitting of the decay profiles to a three-exponential function. Residuals are shown versus time; Figure S8 Changes in the absorption spectra of **Q1** (10 μM), **Q2** (6 μM), and **Q3** (10 μM) over time towards L-Cys and L-GSH (100 μM) in a 0.1 M phosphate buffer with CH_3_CN (20%, *v*/*v*, pH = 7.4); Figure S9 Fluorescence intensity of **Q4** (20 μM) at different pH values in the absence/presence of HSO_3_^−^ (1 mM); Figure S10 ^1^H-NMR spectra of **Q3** in the presence of excess ME (2-mercaptoethanol) in dimethyl sulphoxide-*d_6_*; Figure S11 ESI mass spectrum of **Q3** (10 µM) with 2-mercaptoethanol (1000 µM); Figure S12 Fluorescence intensity of **Q1** (10 µM), **Q2** (6 µM), and **Q3** (10 µM) in a 0.1 M phosphate buffer with CH_3_CN (20%, *v*/*v*, and pH = 7.4) in the presence of biothiols (1 mM), NEM (1 mM); Table S1 The fluorescence lifetimes, quantum yields, and rate constant for radiative and non-radiative decay in acetonitrile.

## Sample Availability

Samples of the compounds **Q1**–**Q4** are available from the authors.
