# Supplementary material for: Comparison of Reactive Sites in 2(1H)-Quinolone Derivatives for the Detection of Biologically Important Sulfur Compounds
Source: Molecules. 2023 Aug 9;28(16):5965. doi: 10.3390/molecules28165965 (PMC10459984; doi:10.3390/molecules28165965)
Supplement: Supplementary file 1 [file molecules-28-05965-s001.zip › molecules-2505128-supplementary.pdf]

Comparison of reactive sites in 2(1*H*)-quinolone derivatives for the detection of biologically important sulfur compounds

Jolanta Kolińska*, Aleksandra Grzelakowska, Marcin Szala, Radosław Podsiadły

Institute of Polymer and Dye Technology, Faculty of Chemistry, Lodz University of Technology, Stefanowskiego 16, 90-537 Lodz, Poland

Corresponding author: jolanta.kolinska@p.lodz.pl

Supplementary Information

Contents

1. **Figs S1-S3** ^{1}H NMR spectra of **Q1-Q3** in DMSO-d_6
2. **Figs S4-S6** HRMS spectra of **Q1-Q3**
3. **Fig. S7** The fluorescence decay profiles of the compounds **Q1-Q4** in acetonitrile with excitation at 376.2 nm. Lamp response was determined using Ludox. The fitting of the decay profiles to a three-exponential function. Residuals are shown versus time
4. **Tab. S1** The fluorescence lifetimes, quantum yields, and rate constant for radiative and non-radiative decay in acetonitrile
5. **Fig. S8** Changes in the absorption spectra of **Q1** (10 μM), **Q2** (6 μM), **Q3** (10 μM) over time towards L-Cys and L-GSH (100 μM) in 0.1 M phosphate buffer with CH_3CN (20%, v/v, pH=7.4)

6. **Fig. S9** Fluorescence intensity of **Q4** (20 μM) at different pH values in the absence/presence of HSO_3^- (1 mM)
7. **Fig. S10** ^{1}H-NMR spectra of **Q3** in the presence of excess ME (2-mercaptoethanol) in dimethyl sulphoxide-d_6
8. **Fig. S11** ESI mass spectrum of **Q3** (10 μM) with 2-mercaptoethanol (1000 μM)
9. **Fig. S12** Fluorescence intensity of **Q1** (10 μM), **Q2** (6 μM), **Q3** (10 μM) in 0.1 M phosphate buffer with CH_3CN (20%, v/v, pH=7.4) in the presence of biothiols (1 mM), NEM (1 mM)

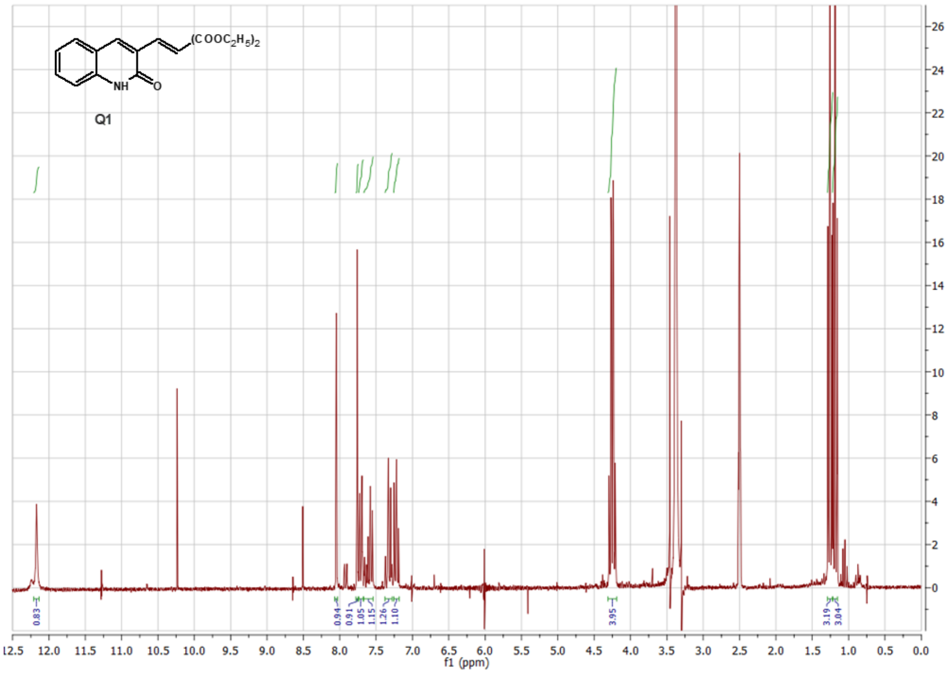

Figure S1 ^{1}H NMR spectrum of **Q1** in DMSO-d_6

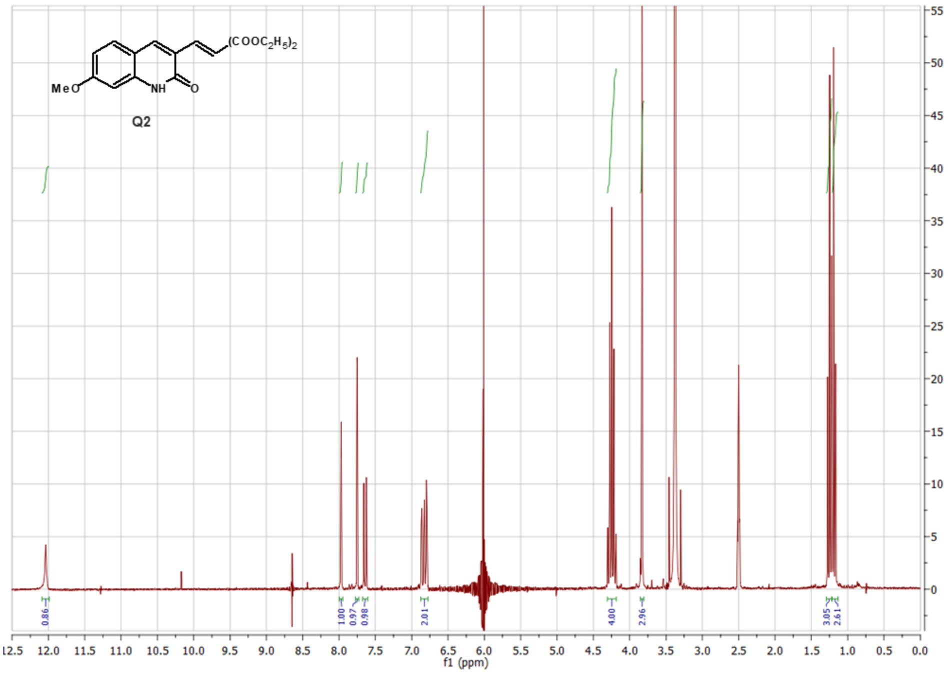

Figure S2 ^{1}H NMR spectrum of **Q2** in DMSO-d_6

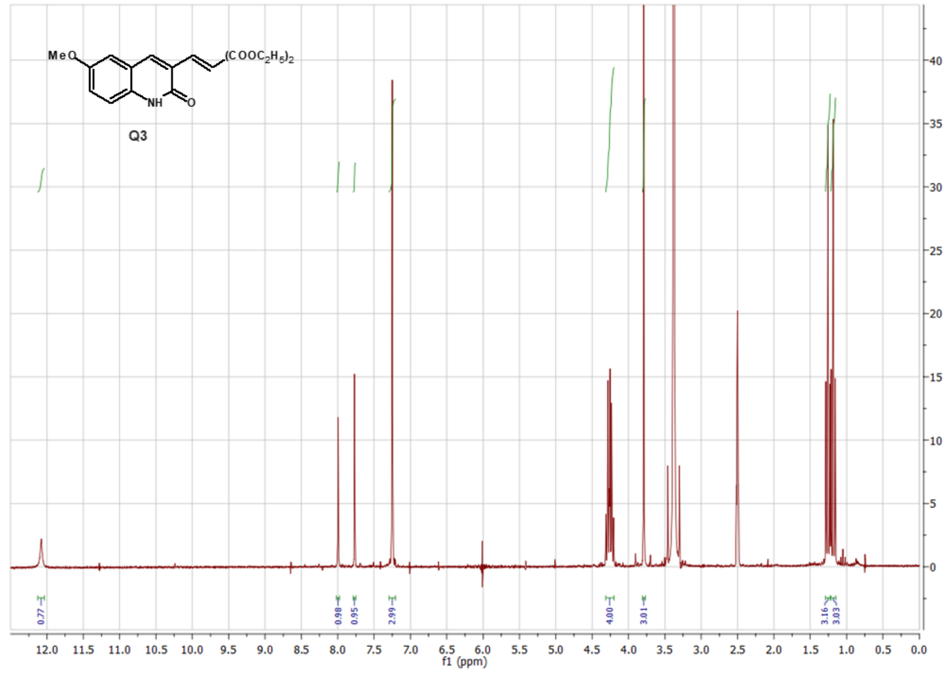

Figure S3 ^{1}H NMR spectrum of **Q3** in DMSO-d_6

Monoisotopic Mass, Even Electron Ions
58 formula(e) evaluated with 1 results within limits (all results (up to 1000) for each mass)
Elements Used:
C: 0-35   H: 0-60   N: 0-1   O: 0-6

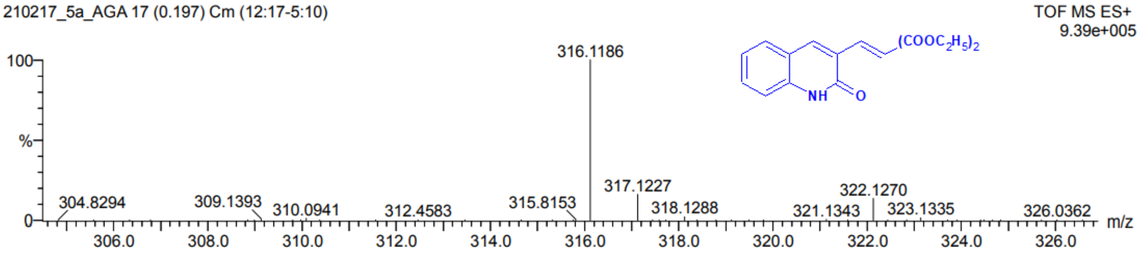

Minimum:				-1.5				
Maximum:		5.0	5.0	80.0				
Mass	Calc. Mass	mDa	PPM	DBE	i-FIT	Norm	Conf(%)	Formula
316.1186	316.1185	0.1	0.3	9.5	193.7	n/a	n/a	C17 H18 N O5

Figure S4 HRMS spectrum of **Q1**

Monoisotopic Mass, Even Electron Ions
121 formula(e) evaluated with 1 results within limits (all results (up to 1000) for each mass)
Elements Used:
C: 0-35   H: 0-60   N: 0-1   O: 0-6   Na: 0-1

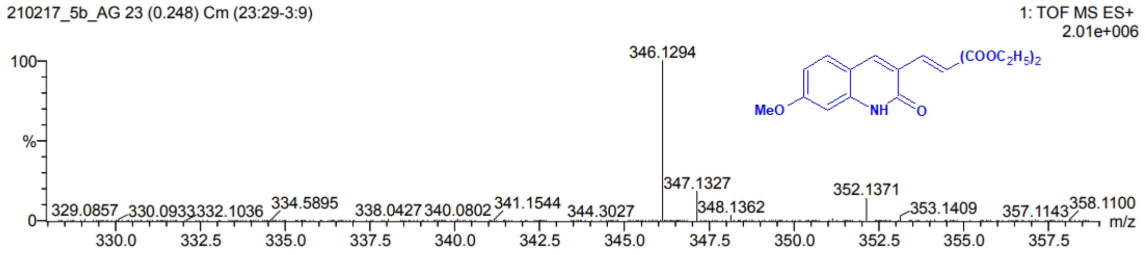

Minimum:				-1.5				
Maximum:		5.0	5.0	80.0				
Mass	Calc. Mass	mDa	PPM	DBE	i-FIT	Norm	Conf(%)	Formula
346.1294	346.1291	0.3	0.9	9.5	952.6	n/a	n/a	C18 H20 N O6

Figure S5 HRMS spectrum of **Q2**

Monoisotopic Mass, Even Electron Ions
121 formula(e) evaluated with 1 results within limits (all results (up to 1000) for each mass)
Elements Used:
C: 0-35   H: 0-60   N: 0-1   O: 0-6   Na: 0-1

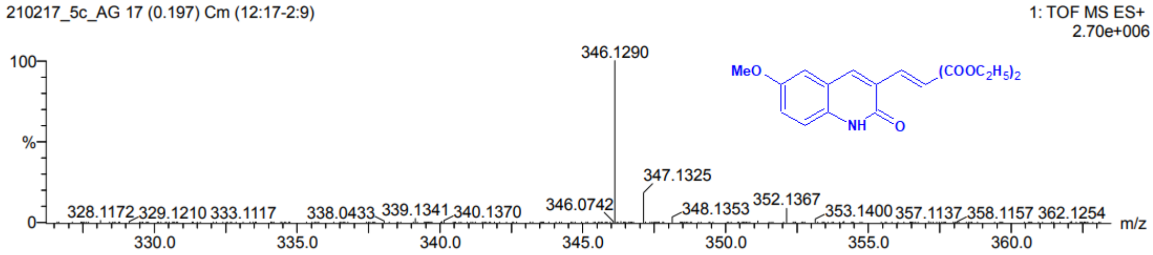

Minimum:				-1.5				
Maximum:		5.0	5.0	80.0				
Mass	Calc. Mass	mDa	PPM	DBE	i-FIT	Norm	Conf(%)	Formula
346.1290	346.1291	-0.1	-0.3	9.5	735.1	n/a	n/a	C18 H20 N O6

Figure S6 HRMS spectrum of **Q3**

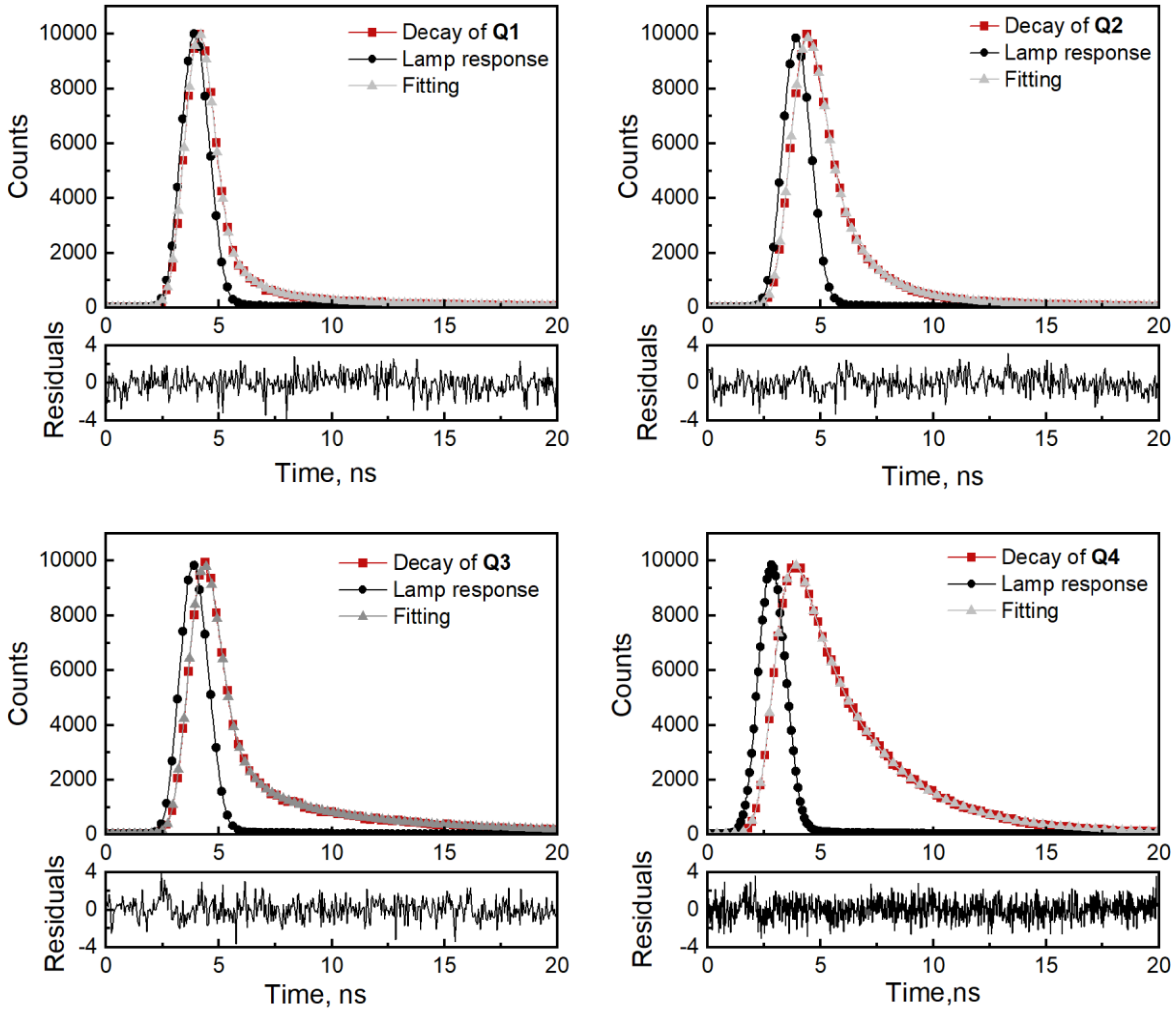

Figure S7 The fluorescence decay profiles of the compounds **Q1-Q4** in acetonitrile with excitation at 376.2 nm. Lamp response was determined using Ludox. The fitting of the decay profiles to a three-exponential function. Residuals are shown versus time

Table S1 The fluorescence lifetimes, quantum yields, and rate constant for radiative and non-radiative decay in acetonitrile

	**Φ_{em} (–)**	**τ (ns)**	**k_r ($10^9 s^{-1}$)**	**k_{nr} ($10^9 s^{-1}$)**	**k_r / k_{nr}**
**Q1**	0.0020	1.25	0.0016	0.7984	0.0020
**Q2**	0.0019	1.86	0.0010	0.5366	0.0019

**Q3**	0.0242	3.09	0.0078	0.3157	0.0248
**Q4**	0.0930	3.12	0.0298	0.2907	0.1025

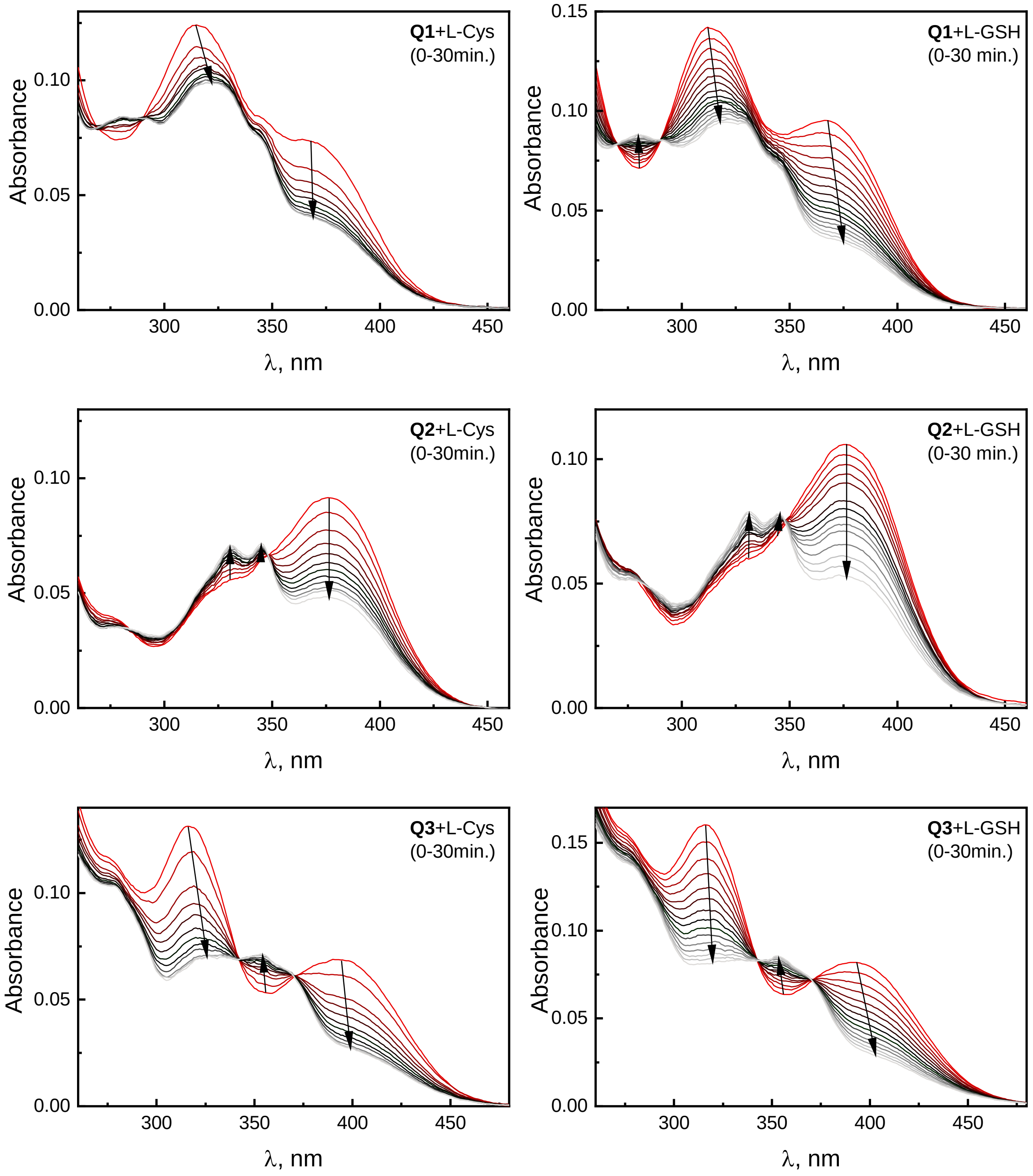

Figure S8 Changes in the absorption spectra of **Q1** (10 μM), **Q2** (6 μM), **Q3** (10 μM)

over time toward L-Cys and L-GSH (100 μM) in 0.1 M phosphate buffer with CH_3CN (20%, v/v, pH=7.4)

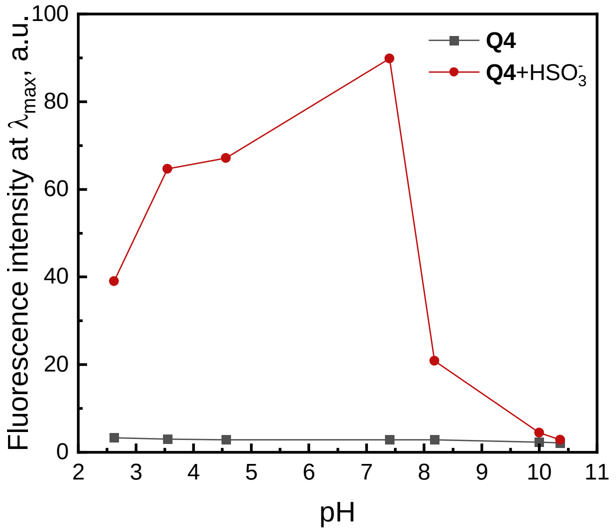

Figure S9 Fluorescence intensity of **Q4** (20 μM) at different pH values in the absence/presence of HSO_3^- (1 mM)

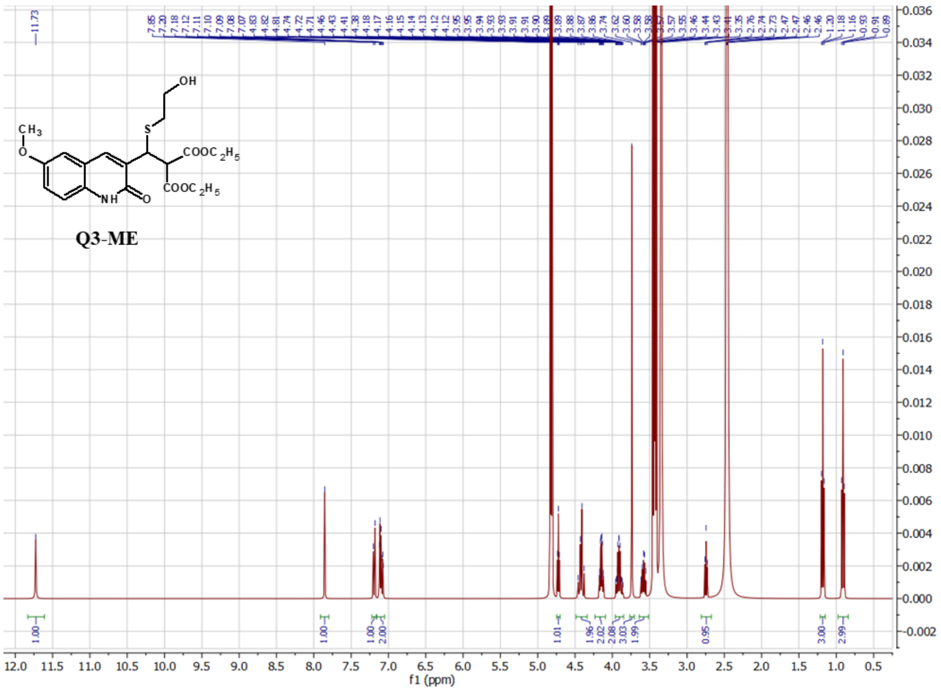

DMSO-*d*6

Figure S10 ^{1}H-NMR spectra of **Q3** in the presence of excess ME (2-mercaptoethanol) in dimethyl sulphoxide-d_6

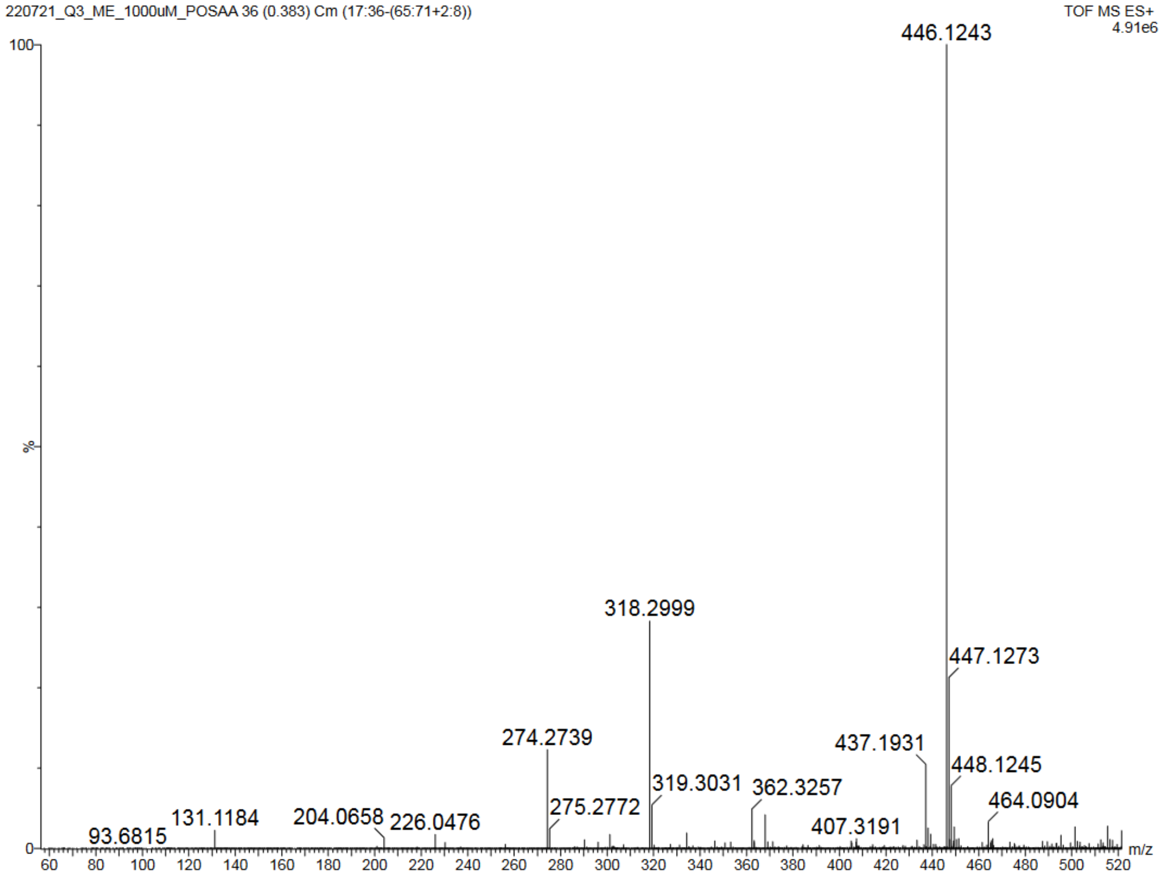

Figure S11 ESI mass spectrum of **Q3** (10 μM) with 2-mercaptoethanol (1000 μM)

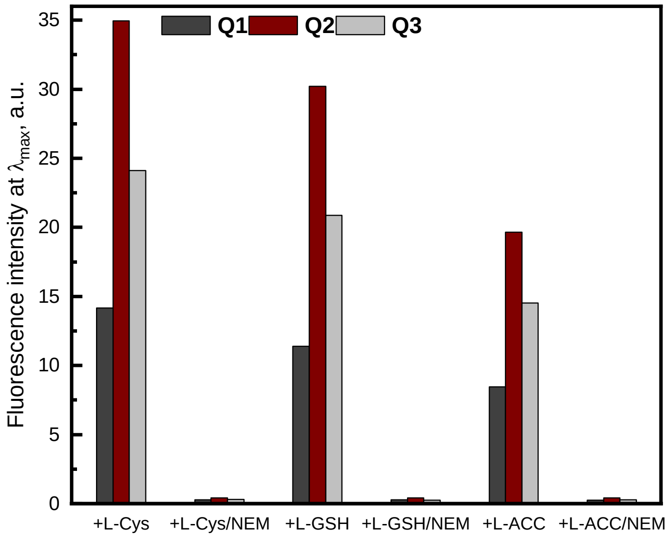

Figure S12 Fluorescence intensity of **Q1** (10 μM), **Q2** (6 μM), **Q3** (10 μM) in 0.1 M phosphate buffer with CH_3CN (20%, v/v, pH=7.4) in the presence of biothiols (1 mM), NEM (1 mM)
